# Sequence features involved in the mechanism of 3' splice junction wobbling

**DOI:** 10.1186/1471-2199-11-34

**Published:** 2010-05-07

**Authors:** Kuo-Wang Tsai, Wen-Ching Chan, Chun-Nan Hsu, Wen-chang Lin

**Affiliations:** 1 Institute of Biomedical Sciences, Academia Sinica, Taipei, Taiwan; 2 Bioinformatics Program, Taiwan International Graduate Program, Academia Sinica, Taipei, Taiwan; 3 Institute of Bioinformatics, School of Medicine, National Yang-Ming University, Taipei, Taiwan; 4 Institute of Information Sciences, Academia Sinica, Taipei, Taiwan; 5 Information Sciences Institute, University of Southern California, Marina del Rey, CA, USA

## Abstract

**Background:**

Alternative splicing is an important mechanism mediating the diversified functions of genes in multicellular organisms, and such event occurs in around 40-60% of human genes. Recently, a new splice-junction wobbling mechanism was proposed that subtle modifications exist in mRNA maturation by alternatively choosing at 5'- GTNGT and 3'- NAGNAG, which created single amino acid insertion and deletion isoforms.

**Results:**

By browsing the Alternative Splicing Database information, we observed that most 3' alternative splice site choices occur within six nucleotides of the dominant splice site and the incidence significantly decreases further away from the dominant acceptor site. Although a lower frequency of alternative splicing occurs within the intronic region (alternative splicing at the proximal AG) than in the exonic region (alternative splicing at the distal AG), alternative AG sites located within the intronic region show stronger potential as the acceptor. These observations revealed that the choice of 3' splice sites during 3' splicing junction wobbling could depend on the distance between the duplicated AG and the branch point site (BPS). Further mutagenesis experiments demonstrated that the distance of AG-to-AG and BPS-to-AG can greatly influence 3' splice site selection. Knocking down a known alternative splicing regulator, hSlu7, failed to affect wobble splicing choices.

**Conclusion:**

Our results implied that nucleotide distance between proximal and distal AG sites has an important regulatory function. In this study, we showed that occurrence of 3' wobble splicing occurs in a distance-dependent manner and that most of this wobble splicing is probably caused by steric hindrance from a factor bound at the neighboring tandem motif sequence.

## Background

Alternative splicing is an important mechanism of gene regulation in the human genome, occurring in around 40-60% of human genes [1]. The 5' and 3' alternative splicing events contribute 25% of all alternative splicing instances and such events often result in frameshift mutations or insertion/deletion of amino acids in the expressed proteins [2]. Recent studies indicated that certain alternative 5'/3' tandem splice site selections are only a few nucleotides apart, and that such short nucleotide length variations can still lead to subtle changes in protein structure through the modification of coding amino acids [3-5]. Interestingly, this phenomenon occurs throughout the genome, which could result in many protein isoforms with one amino acid insertion or deletion [6-15]. Splicing at junctions that contain GTNGT at the 5' splice site or NAGNAG at the 3' splice site would generate long transcripts (3 bp included) or short transcripts (3 bp excluded) during the wobble splicing process [7,9,16]. Although tandem motifs are common in human genes, only a small fraction of them can produce wobble splicing isoforms (GTNGT: 2%; NAGNAG: 16%) [7,9-11]. Therefore, a complicated mechanism could exist for regulating tandem splice site choice during the wobble splicing process. Previous studies have shown that the high fidelity of splice site recognition involves specific networks of RNA-protein, protein-protein and RNA-RNA interactions [17-19]. The mechanism of acceptor site choice requires more complicated control than for donor sites, because the splicing factors interact flexibly with cis-elements such as branch point sequences (BPS), polypyrimidine tracts and the acceptor AG site. In our previous studies, we found that selection of acceptor sites in 3' wobble splicing could be affected by (i) a tandem splice site (NAGNAG) and (ii) component sequences occurring between the BPS and the NAGNAG [including BPS and polypyrimidine tracts (PPTs)] [12]. Interestingly, we also found that mutations or single nucleotide polymorphisms (SNPs) at the BPS can disturb 3' wobble splicing selections by creating an aberrant branch point [12]. This would affect the BPS-to-AG nucleotide distance and hence alter the wobble splicing selection pattern. However, the detailed mechanisms for such wobble splicing choices are currently unclear and remain to be studied. In this study, we utilized a minigene approach to demonstrate that the distance between two tandem splice sites or between BPS and AG plays an important role in 3' alternative splicing choice at nearby tandem splice sites, and that this phenomenon is indiscriminate.

## Results

### Occurrence of wobble splicing at tandem splice sites separated by a short distance

**Figure 1:**
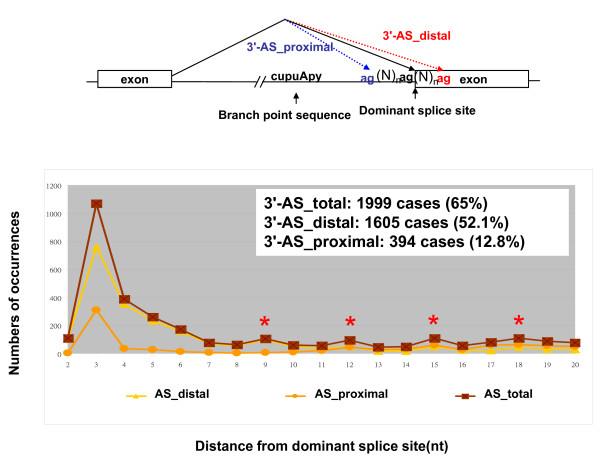
**Distribution of 3' alternative splicing at positions ranging from two to twenty nucleotides from the dominant splice site**. Schematic representation of three groups of 3' alternative splicing occurring close to the dominant site: 3'-AS_dominant site (black line), 3'-AS_proximal site (blue dashed line) and distal site (red dashed line). The brown squares indicate the total number of alternative splices at the 3' splice sites. Alternative splicing at the proximal splice site (intronic splice site) and the distal splice site (exonic splice site) is indicated by triangles and circles. The numbers of each of the three groups of alternative splicing occurring within six nucleotides of the dominant splice site are indicated in the top panel and the percentage in each group was determined as (number of alternative splices within six nucleotides of the dominant site)/(total number of alternative splices within 20 nucleotides of the dominant splice site) × 100. The red asterisks indicate that alternative splicing occurred at in-frame sites.

**Figure 2:**
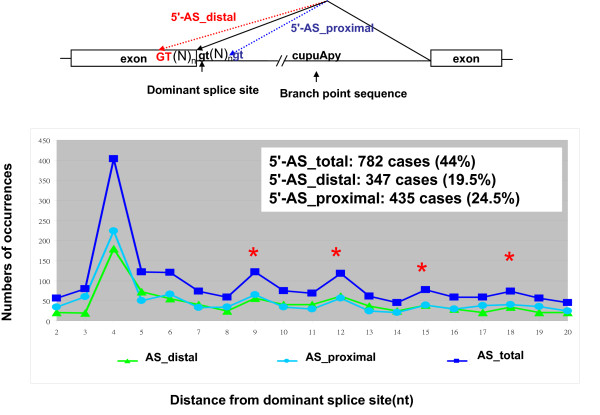
**Distribution of 5' alternative splicing at positions ranging from two to twenty nucleotides from the dominant splice site**. Schematic representation of three groups of 5' alternative splicing occurring close to the dominant site: 5'-AS_dominant site (black line), 5'-AS_proximal site (blue dashed line) and distal site (red dashed line). The blue squares indicate the total number of alternative splices at the 5' splice sites. Alternative splicing at the proximal splice site (intronic splice site) and the distal splice site (exonic splice site) is indicated by triangles and circles. The numbers of each of the three groups of alternative splicing occurring within six nucleotides of the dominant splice site are indicated in the top panel and the percentage in each group was determined as (number of alternative splices within six nucleotides of the dominant site)/(total number of alternative splices within 20 nucleotides of the dominant splice site) × 100. The red asterisks indicate that alternative splicing occurred at in-frame sites.

**Figure 3:**
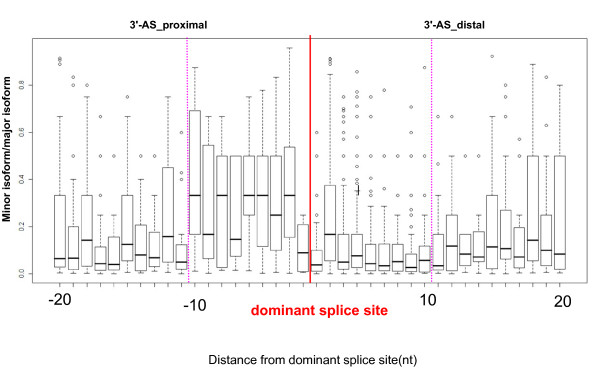
**The ratios of ESTs between 3'-AS at major and minor sites was plotted respective to their positions**. The Y-axis indicates the distribution of ratio of ESTs between major and minor sites. The X-axis denotes the nucleotide distance between alternative and dominant splice sites. Alternative 3' splice sites upstream from the dominant splice site (red line) denote as negative, those downstream are positive. Pink dashed line denotes the alternative splices within ten nucleotides of the dominant site.

GTNGT- and NAGNAG-based wobble splicing events are widespread in the human genome, especially the 3' wobble splicing event that, according to an expressed sequence tag (EST) database survey, occurs in 30% of human genes and is active in at least 5% of genes [7]. In order to investigate further the distribution of wobble splicing events within the human genome, we have performed an in-depth computational analysis using a well known database--the Alternative Splicing Database (ASD) [20]. The ASD is derived from EST entries and reports the use of alternative splicing sites within the human transcriptome. In this study, we extracted 7,400 and 8,223 explicit alternative sites in 5' and 3' junctions, respectively, removing all ambiguous and conflicting instances. In the analysis of wobble splicing usage, the splice sites used for generating the highest numbers of transcripts according to the ASD dataset were defined as dominant splice sites. Additional putative alternate splice sites located around dominant splice sites are defined as proximal sites and distal sites as indicated in Figure 1 and 2. In each splicing instance, the number of ESTs occurring in relation to the distance from the dominant splice site was tabulated as shown in Figure 1 and 2. Our data indicated that short-distance wobble splicing events at the 3' tandem NAGNAG motif occur with a higher frequency than 5' GTNGT sites (1,999 cases vs. 782 cases), because the 3' end of an intron has a more intricate set of regulatory elements (Figure 1 and 2). Interestingly, 3' alternative splicing at a tandem acceptor has a higher frequency if the distance between the two tandem splice sites is less than six nucleotides. The frequency decreases significantly when the distance from the dominant splice site increases (> 6 nucleotides). However, alternative acceptor choice occurring within six nucleotides of the dominant splice site is more frequent in the exonic region (3'-AS_distal) than the intronic (3'-AS_proximal) region (3'-AS_distal: 1,605 cases; 3'-AS_proximal: 394 cases) (Figure 1). This is probably because the PPT preceding AG is a required feature for the splicing process; this therefore reduces the occurrence of 3'-AS_proximal site choices. Surprisingly, we have observed a higher ratio of minor isoforms in the 3'-AS_proximal AG selection dataset than in the 3'-AS_distal AG dataset even though the alternative AG is closer to the dominant AG (AG-to-AG distance < 10 nucleotides) (Figure 3). Although we observed a lower frequency of alternative splicing within the intronic region (3'-AS_proximal AG) than in the exonic region (3'-AS_distal AG), the alternative AG in the intronic region does possess greater potential as an acceptor site. Presumably, the spliceosome complex recognizes the branch point and scans downstream for the first AG by the hypothesized linear scanning mechanism [21-24]. The above results indicated that the process of 3' splice site selection may depend on the distance between the proximal AG and the distal AG (AG-to-AG), or the BPS and tandem splice sites (BPS-to-AG). Interestingly, we did not observe this feature involved in 5' short-distance wobble splicing. According to the ASD screening data, only one high frequency of 5' alternative splicing occurs at four nucleotides upstream or downstream from the dominant splice site [GT(N)_2 _GT: 403 cases] (Figure 2). Previous studies indicated that such bias could result from the strong U1 snRNP-binding conserved sequence at 5' splice sites [9,25]. We further analyzed the distribution of 5'-AS in UTR and CDS and found that 5'-GT(N)_2_GT alternative splicing frequently occurs in UTR region (~ 50%) (Additional file 1, Figure S1). While wobble splicing occurred in UTR region, it would not alter open reading frame and could escape from NMD degradation. Therefore, it is a possible explanation for overabundance of the 5'-GT(N)_2_GT wobble splicing.

### Slu7 did not affect 3' wobble splicing at tandem motif sites

**Figure 4:**
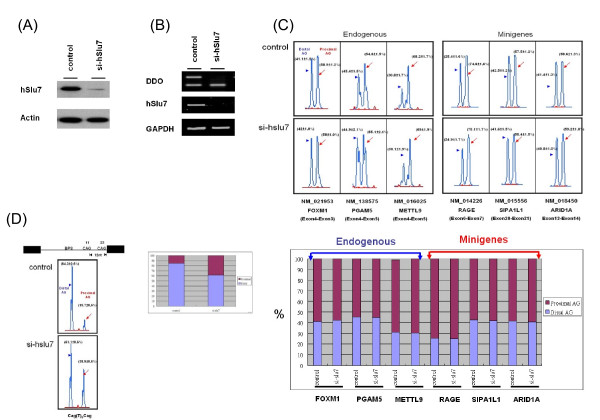
**The effect of hSlu7 on tandem splice site selection**. HeLa cells were transfected with the RNAi oligonucleotide directed against either hSlu7 or luciferase (control). (A) After 48 h, total protein was extracted, separated by 10% SDS-PAGE, transferred to a membrane and probed for hSlu7 and actin. (B) The total RNA was collected, followed by RT-PCR using specific primers. The alternative splicing of the DDO-1 gene was affected by the hSlu7 protein concentration (positive control). GAPDH, a housekeeping gene, was amplified in each sample to confirm that approximately the same amount of cDNA was used for each reaction. (C) and (D) Analysis of 3' wobble splicing of three endogenous genes (FOXM1: NM_021953, PGAM5: NM_138575 and METTL9: NM_016025) and four minigenes (RAGE: NM_014226, SIPA1L1: NM_015556, ARID1A: NM_018450 and AG(T)_9_CAG) in hSlu7 knockdown HeLa cells using capillary electrophoresis (upper panel). The relative percentage of the two isoforms was calculated using GeneScan 3.7 (lower panel).

The *cis *elements and *trans*-acting factors are involved in precise recognition of the splice sites during the splicing process [26]. Therefore, we first investigated whether *trans*-acting splicing factor was involved in 3' wobble splicing. The splicing factor Slu7 has been shown to affect 3' AG selection during step II of the splicing process *in vitro *and has been suggested to affect alternative splicing choice *in vivo *[19,24,27]. To determine whether the *trans *protein factor hSlu7 is involved in 3' splice site selection in wobble splicing events, we used an RNAi approach to knockdown hSlu7. hSlu7 RNAi treatment led to a 90% reduction in hSlu7 protein level and affected the exon inclusion/skipping ratio of a target gene, D-aspartate oxidase (*DDO*), changing it from mostly exon skipping to inclusion. This result indicated that as expected, the RNAi-mediated reduction in the nuclear level of hSlu7 had functional consequences in alternative splice choice (Figure 4A and 4B). However, the patterns of 3'-NAGNAG-based wobble splicing were not significantly altered by hSlu7 knockdown (Figure 4C), which suggests that hSlu7 is dispensable for 3'-NAGNAG-based alternative splicing in very closely linked tandem motifs (within three nucleotides). This is observed in endogenous genes as well as in transfected minigene constructs. Previous studies showed that aberrant AG site selection could be demonstrated by *in vitro *splicing assay using Δ*hSlu7 *extracts, while the duplicated AG is located upstream or downstream of the normal AG (AG-to-AG distance: from 6 to 12 nucleotides) [27]. To further determine whether the distance between AG-to-AG is a critical factor in slu7 deciding 3' splice-site choice and the efficiency of our hSlu7 RNAi treatment, we examined the same AG(N)_9_CAG construct used from previous publication (11AG/23AG construct) [27]. As shown in Figure 4D, the use of the distal AG site (23AG) was decreased and proximal AG site (11AG) was significantly activated by reducing hSlu7 protein level in si-hSlu7 transfected cells. This result confirmed that the AG site choice modulation effects of hSlu7 and appropriated distance between duplicate AGs might be needed for 3' splice sites selection by the Slu7 protein. From our bioinformatic and experimental results, the very short distance (three nucleotides) between proximal and distal AG sites might not be modulated by Slu7 alone.

### Tandem splice site-based wobble splicing depends on the AG-to-AG and BPS-to-AG distance

**Figure 5:**
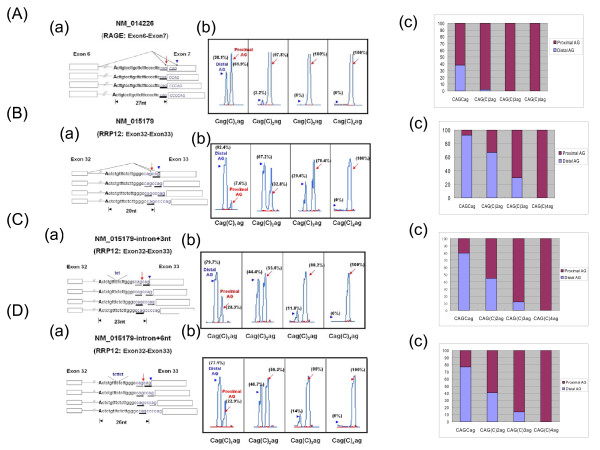
**3' acceptor site selection depends on the AG-to-AG and BPS-to-AG distances**. NM_014226-E6-I6-E7 (RAGE) (A) and NM_015179-E32-I32-E33 (RRP12) (B) minigene vectors containing four different distances between the proximal and distal AG, CAG(C)_1_AG, CAG(C)_2_AG; CAG(C)_3_AG and CAG(C)_4_AG (construction as shown in a panels). These minigenes were transfected into a HeLa cell line, and after 48 h total RNA and alternative splicing patterns were assayed as mentioned in the Materials and Methods. The expression profiles of these minigenes are indicated in the b panels and the relative percentage of the two isoforms was showed in the c panels. (C) and (D) The BPS-to-AG distance of NM_015179-E32-I32-E33 was extended by inserting cytosines into the PPT region. These constructs are shown in the a panels and their expression profiles are indicated in the b panels and the relative percentage of the two isoforms was showed in the c panels. The arrowheads and arrows indicate the use of distal and proximal AG, respectively. The percentage of wobble splicing isoforms is shown at the top of each b panel and the relative use of each AG is indicated by the thickness of the underlining in each a panel.

Next, we determined whether the physical AG-to-AG and BPS-to-AG distance was involved in splice site selection. We constructed two minigene constructs both containing strong 3' wobble splicing tandem motifs (CAGCAG). According to EST information, one gene (RAGE: NN_014226) preferentially uses the proximal AG site (proximal/distal: 61.9%/38.1%) and another gene (RRP12: NM_015179) preferentially uses the distal AG site (proximal/distal: 7.6%/92.4%). As shown in Figure 5A and 3B, we inserted cytosine residues to increase the distance between proximal AG and distal AG. This makes the proximal AG become more competitive than the distal AG sites. Gradually increasing the number of cytosine residues to four in two minigenes caused the proximal AG to be used almost exclusively. Such results support the previous observation that nucleotide distance between proximal and distal AG affects the wobble splicing choice (Figure 5A and 5B). A short distance of six nucleotides or less could create competition between distal and proximal AG sites (Figure 5A and 5B). Next, we further examined the AG site choice of the NM_015179 minigene by increasing the BPS-to-AG distance. These minigene constructs with varied BPS-to-AG distances were introduced into HeLa cells and splicing patterns were analyzed by a capillary electrophoresis approach. As shown in Figure 5B, C and 5D, increasing the BPS-to-AG nucleotide number could significantly reinforce the selection of the proximal AG splice site. When the distance between tandem AG splice sites is reduced to less than five nucleotides, the proximal AG cannot completely compete with the distal AG because of the increased BPS-to-AG distance (Figure 5B, C and 5D). This implies that the BPS-to-AG distance can also affect splice site selection, but also that it cannot influence the occurrence of wobble splicing at close tandem motifs. In conclusion, the choice of tandem acceptor sites during wobble splicing is under the influence of both AG-to-AG and BPS-to-AG distance.

## Discussion

Previous studies have shown that the high fidelity of splice site recognition involves specific networks of RNA-protein, protein-protein and RNA-RNA interactions [17-19]. However, the detailed mechanisms for alternative splicing at tandem motifs are currently unclear. Previous studies indicated that such tandem motifs at splicing junctions are common in human genes, but only a small fraction of them can generate wobble in splicing selection [7,9-11]. Our data confirmed that a high frequency of 5' wobble splicing events is located at four nucleotides from the dominant donor site and this is hypothesized to be associated with the binding affinity of U1 snRNA [9,25]. Based on this, 5' wobble splicing could occur when one donor site is effectively competing with the other donor site for U1 binding. Alternatively, 3' alternative wobble splicing seems to occur more frequently at closely associated tandem acceptor sites (< 6 nucleotides). Thus, the choice between 3' acceptor sites appears to be more complicated than that of 5' donor sites. It is possible that splicing factors flexibly interact with cis-elements, such as BPS, PPTs and AG splice sites, during 3' splicing. According to the proposed linear scanning mechanism model [21-24], the spliceosome recognizes the branch point and scans downstream for the first AG. However, in this study we also observed many instances in which use of the distal AG was preferred, which could not be explained by the scanning model alone.

RNA surveillance, also known as nonsense-mediated mRNA decay (NMD), is an mRNA quality-control mechanism that degrades abnormal mRNAs such as misspliced mRNA transcripts [28]. By recognizing mRNAs containing a premature termination codon, NMD eliminates the production of the truncated protein encoded by the misspliced transcripts that could function to the detriment of cells [29]. Putative splicing sites located close to the dominant splice site may cause wobble splicing, resulting in small insertion/deletion changes in transcripts [9,12,25,30]. Our data also showed that NMD is involved in distribution of short-distance alternative splicing. Figure 1 and 2 show that while most of the wrongly spliced mRNA transcripts were degraded by NMD, those wobble spliced at tandem motifs, AG(N)_n_AG or GT(N)_n_GT (n = 1, 4, 7, 10, 13 or 16), might have a better chance to escape from the NMD surveillance because of the occurrence of an in-frame insertion/deletion of one or two amino acids in these mRNAs without generating a premature stop codon. Frame-shifting a tandem splice site (n = 0, 2 or 3) has severe consequences for protein function because of the creation of altered protein residues or loss of mRNA transcripts by the wobble splicing process. One high frequency 5' wobble splicing is located four nucleotides from the dominant donor site, and most of the frame-shifting transcripts would be disrupted by NMD. In contrast to 5' alternative splicing, a high frequency of 3' alternative splicing occurs at ± three nucleotides (635 cases), which can increase protein diversity by altering 1-2 amino acids. Although this only subtly changes the protein sequence, it might influence protein function, for example in NR3C1, DRPLA, PAX3, PAX7, IGF1R and ING4 [10,31-34]. Previous studies indicated that four wobble-splicing isoforms of ING4 differ in several functional aspects including protein localization, protein degradation, protein-protein interactions, transcriptional activity and cell spreading and migration [13,35,36]. Moreover, alternative splicing at a 5' or 3' tandem splice site may play an important role in the progression of disease, because reported cases include human genes *WT1 *and *ABCA4 *[37,38].

Traditionally, alternative splicing is expressed in a tissue type or developmental stage-dependent manner through regulating certain splicing factors. However, most NAGNAG- or GTNGT-based wobble splicing events did not show differential expression patterns of spliced isoforms in various tissues. Only a small fraction of these were reported to be tissue specific in genes including *ITGAM*, *SMARCA4 *and *BTNL2 *[7]. In this study, hSlu7 failed to alter short-distance wobble splicing, which may be due to the close distance (< 6 nucleotides) between proximal and distal AG sites. Therefore, the *trans*-splicing factor, hSlu7, might not be involved in the recognition of proximal and distance splice sites in NAGNAG-based wobble splicing. According features of the neighboring nucleotides around tandem splice sites and G/C content of PPT, Shina *et al*. successfully developed a method to accurately predict of NAGNAG-based wobble splicing pattern. In this study, we revealed that distance of AG-to-AG and BPS-to-AG both influenced choice of tandem acceptor sites during 3'-short-distance wobble splicing. Based on these observations, we believe that most of this wobble splicing is most likely caused by steric hindrance from a factor bound at the surrounding tandem motif sequence. Based on this hypothesis, wobble splicing could be predicted according to *cis*-element sequence features as reported [39,40].

## Conclusion

In summary, this study supplies further evidence of the involvement of acceptor site selection in wobble splicing at close tandem splice sites. Overall, our data reveal that the mechanism of short-distance 3' wobble splicing is stochastic and depends on the BPS-to-AG and AG-to-AG distance.

## Methods

### Frequency of 3' and 5' alternative splicing

In this study, all investigations were based on the third release of human 36.35i from the ASD http://www.ebi.ac.uk/asd/. For the analysis of the human 5' and 3' alternative splicing, the splicing events data file (AltSplice-rel3.events.txt) and gene sequence file (AltSplice-rel3.genes.txt) were downloaded from the ASD. The interesting intron splicing events were extracted from the isoform, and thus were classified according to their location 5' or 3' to introns. A total of 19,874 intron isoform events were reported in the ASD, which comprised 8,772 and 9,491 distinct alternative sites in 5' and 3', respectively. In each of the instances of splicing, the splice site used to produce the major transcripts was defined as the dominant splice site. An alternative site with equal EST support was identified as an ambiguous case. An alternative site with a majority of disagreement among all corresponding II events was treated as a conflicting case. After filtering all ambiguous and conflicting cases, 7,400 and 8,223 explicit alternative sites remained in 5' and 3', respectively. For each of these events, the number of ESTs that occurred was recorded in relation to the distance from the dominant splice site.

### Plasmid constructs

The genomic DNA of NM_014226-Exon 6-7 and NM_015179-Exon 32-33 was amplified by PCR using primer pair NM_014226-F/R and NM_015179-F/R from genomic DNA of the AZ-521 cell line. The amplified fragments were cloned into pGEM-T easy vector (Promega). After determining their sequence by an autosequencer, a minigene construct was generated by subcloning the genomic DNA of NM_014226-Exon 6-7 and NM_015179-Exon 32-33 into the EcoRI site of the pEGFP-C1 vector (Clontech). The minigenes containing various AG-to-AG or BPS-to-AG distances were generated by overlapping PCR as follows. The plasmid human minigene was used as template for a first PCR with a variant AG-to-AG distance primer set or BPS-to-AG distance primer set and the PCR products were used as a megaprimer. A second PCR was performed using the original minigene as template and the product subcloned into pGEM-T easy vector. After confirming the sequence, we subcloned the amplified product into pEGFP-C1 expression vector. The specific PCR primer pairs are listed in Additional file 2, Table S1. The 11AG/23AG plasmid construct is provided by Dr. R. Reed and subcloned into pRGFP-C1 expression vector.

### Splicing analysis *in vivo*

The minigene plasmids were introduced into HeLa cells by Lipofectamine 2000 (Invitrogen) according to the methods provided by the manufacturer. At 48 h posttransfection, total RNA was extracted, and reverse transcription was carried out using 2.5 μg of poly(A)(+) RNA, oligo-(dT)15 and SuperScript II reverse transcriptase (Invitrogen). The splicing products were analyzed by capillary electrophoresis using the FAM-labeled primer set described in Additional file 2, Table S1.

### Western blotting assay

HeLa cells were transfected with either of the RNAi oligonucleotides (5'-UUCAGAUCCCUUGUCAUAGGCUUCC-3') directed against hSlu7, and random sequence siRNA oligonucleotides (Invitrogen) were used as a negative control. Forty-eight hours after transfection, whole-cell extracts were obtained, subjected to SDS-PAGE and immunoblotted using hSlu7 (sc-10828, Santa Cruz Biotechnology) and anti-actin antibody (sc-1616, Santa Cruz Biotechnology).

### Capillary electrophoresis analysis

PCR reactions were performed in a 20 μl final volume, including 10× PCR buffer, FAM-labeled primer pairs, dNTPs and Takara Taq DNA polymerase (Takara Shuzo Company, Shiga, Japan). PCR conditions were as follows: 94°C for 5 min; 26 cycles at 94°C for 1 min, 58°C for 1 min, 72°C for 1 min; 72°C for 10 min and cooling at 4°C. One microliter of the PCR mix was diluted to 10 μl with formamide (Applied Biosystems, Foster City, CA, USA), containing 1 μl ROX 350 fluorescent size standards (Applied Biosystems), denatured at 95°C for 5 min and cooled at 4°C. Amplified PCR products were separated by an ABI 3100-Avant DNA analyzer using Polymer 3100 POP4, then quantified with GeneScan 3.7 software. The ratio of the wobble splicing isoforms was determined by dividing the peak area of the individual forms by the total area.

## Authors' contributions

KWT performed the main experiments and prepared the manuscript. WCC and CNH performed computational analyses. WCL was responsible for the experimental design and manuscript preparation. All authors read and approved the final manuscript.

## Acknowledgment

We are grateful to Dr R. Reed for the 11AG/23AG construct. This work was supported by research grants from Academia Sinica.

## Supplementary Material

Additional file 1 **Distribution of 5' alternative splice sites relative to dominant splice site**. The brown squares indicate the total number of alternative splices at the 5' splice sites. Alternative splicing occurred in the coding region or in the UTR region of the gene is indicated by green triangles and blue circles respectively. Click here for file

Additional file 2 **Sequences of the oligonucleotides used in this study**. The gene specific PCR primer pairs and FAM-labeled primer pairs are listed in this table. Click here for file
