# Supplementary figures and images for: Sequence features involved in the mechanism of 3' splice junction wobbling
Source: BMC Mol Biol. 2010 May 7;11:34. doi: 10.1186/1471-2199-11-34 (PMC2875228; doi:10.1186/1471-2199-11-34)

## Slide 1
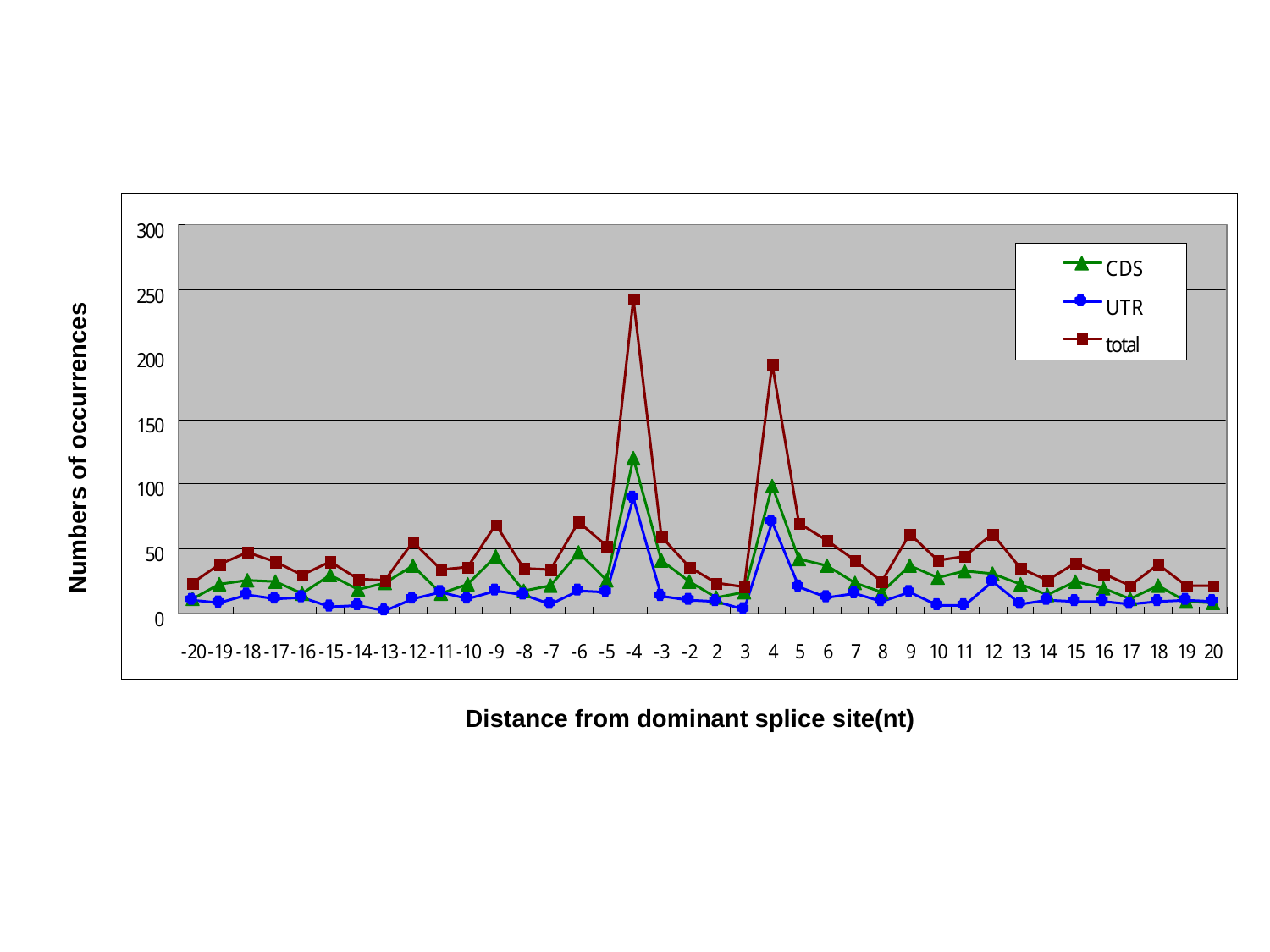

Numbers of occurrences
Distance from dominant splice site(nt)

Supplement: Additional file 1 — Distribution of 5' alternative splice sites relative to dominant splice site. The brown squares indicate the total number of alternative splices at the 5' splice sites. Alternative splicing occurred in the coding region or in the UTR region of the gene is indicated by green triangles and blue circles respectively. [file 1471-2199-11-34-S1.PPT]
