# Supplementary material for: Sequence features involved in the mechanism of 3' splice junction wobbling
Source: BMC Mol Biol. 2010 May 7;11:34. doi: 10.1186/1471-2199-11-34 (PMC2875228; doi:10.1186/1471-2199-11-34)
Supplement: Additional file 2 — Sequences of the oligonucleotides used in this study. The gene specific PCR primer pairs and FAM-labeled primer pairs are listed in this table. [file 1471-2199-11-34-S2.DOC]

| **supplementary table S1 The sequences of the oligonucleotides used in this study** | |
| --- | --- |
| **Specific primers used to PCR-based capillary electrophoresis analysis** | |
| **NM_015179-F** | **5`-FAM-CTGGAGGCTCTGGCATT-3`** |
| **NM_015179-R** | **5`-CTGCGGTTGAGCTTGCTT-3`** |
| **NM_014226-F** | **5`-FAM-AAATGGAATCTTTCACAGAGA-3`** |
| **NM_014226-R** | **5`-TGGCAATCTCGTAGAACAC-3`** |
| **NM_015556 -F** | **5`-FAM-CCAGAGAGCCTCATTTTTTG-3`** |
| **NM_015556-R** | **5`-CTTCAAATCTTCCCGAGG-3`** |
| **NM_018450-F** | **5`-FAM-ATGGCAGAAGGAGGAGAC-3`** |
| **NM_018450-R** | **5`-ATTTGGCTCATAGGACATG-3`** |
| **NM_021953-F** | **5`-FAM-AGCCCTTTGCGAGCAGAAAC-3`** |
| **NM_021953-R** | **5`-GCTGCTCCAGGTGACAATTC-3`** |
| **NM_138575-F** | **5`-FAM-GTCTCATTGGAAGCCGGAAG-3`** |
| **NM_138575-R** | **5`-TGCACACGATGTAGCGGATG-3`** |
| **NM_016025-F** | **5`-FAM-AGTGTCTTGGAGCCAACTAGAG-3`** |
| **NM_016025-R** | **5`-CTTCAGGCAGACTATTCACTTG-3`** |
| **EGFP-F** | **5`- FAM-TAC AAG TCC GGA CTC AGA-3`** |
| **modified primer:** |  |
| **NM_015179-F1** | **5`-GAATACAAGGCCAAGGTGAGGAGGGGGCGGGGC-3`** |
| **NM_015179-R1** | **5`-GCCCCGCCCCCTCCTCACCTTGGCCTTGTATTC-3`** |
| **NM-015179-c-F** | **5`-CTGGGCCAGcCAGAAAGCAAAA-3`** |
| **NM-015179-cc-F** | **5`-CTGGGCCAGccCAGAAAGCAAAA-3`** |
| **NM-015179-ccc-F** | **5`-CTGGGCCAGcccCAGAAAGCAAAA-3`** |
| **NM-015179-I3-F** | **5`-CTGACTCTGTCTTTTCTCTGGGCC-3`** |
| **NM-015179-I6-F** | **5`-CTG ACTCTGTCTCTCTTTCTCTGGGCC-3`** |
| **RT-PCR primer** | |
| **GAPDH-1F** | **5`-** **TGGTATCGTGGAAGGACTCA-3`** |
| **GAPDH-2R** | **5`-** **AGTGGGTGTCGCTGTTGAAG-3`** |
| **DDO1-F** | **5`-** **ATA CACCCATTCACACGCAGA -3`** |
| **DDO1-R** | **5`-** **CAAGGCCTGAACAGTTGACCA-3`** |
| **hslu-7-F** | **5`-** **ATGTCAGCCACAGTTGTAA-3`** |
| **hslu-7-F** | **5`-** **CTGTCCAAGGAAAGAGG-3`** |
